# Q Fever Wildlife Reservoir

**DOI:** 10.3201/eid1105.041272

**Published:** 2005-05

**Authors:** Miguel G. Madariaga

**Affiliations:** * University of Nebraska Medical Center, Omaha, Nebraska, USA

**Keywords:** Madariaga, Miguel, University of Nebraska Medical Center, Infectious Disease

**To the Editor:** To the list of zoonotic infections with wildlife sources reported by Kruse et al. (1), I would add *Coxiella burnetii* infection because of its global impact, extensive presence in the animal kingdom, and potential for use as an agent of bioterrorism (2). *C. burnetii* causes Q fever, a self-limited disease that usually appears as undifferentiated fever, pneumonia, or hepatitis, but which may progress into chronic disease, especially endocarditis, among susceptible persons. Q fever is endemic worldwide in domestic mammals, especially ungulates (cattle, sheep, and goats), but also has been found in wild mammals, birds, and arthropods. The transmission of Q fever to humans from wild rabbits was documented in the 1980s (3). More recently, a study showed seroprevalence of Q fever ranging from 7% to 53% in brown rats (*Rattus norvegicus*) in Oxfordshire, which suggests that they are a possible reservoir for *C. burnetii* in the United Kingdom. The study also speculated why cats, as frequent predators of rats, are important in maintaining the transmission cycle of the disease (4).

A case-control study published in 2001 (5) attempted to define the risk factors for an increase in the incidence of Q fever in French Guiana in 1996. The study found no link between Q fever and domestic ungulates, the usual source of outbreaks. The role of pets, basically dogs and cats, as a reservoir was also excluded. Multivariate analysis showed that living in close proximity to the forest, exposure to wild animals (including bats), and working in public trade or public works were all associated with infection. A strong correlation between large amounts of rainfall and higher incidence of Q fever was found also. All of these findings suggested a wild reservoir as a potential source of the epidemics, although the researchers could not identify a particular species as the specific source.
